# Role of Nitric Oxide in the Cardiovascular and Renal Systems

**DOI:** 10.3390/ijms19092605

**Published:** 2018-09-03

**Authors:** Ashfaq Ahmad, Sara K. Dempsey, Zdravka Daneva, Maleeha Azam, Ningjun Li, Pin-Lan Li, Joseph K. Ritter

**Affiliations:** 1 Department of Pharmacology and Toxicology, Virginia Commonwealth University School of Medicine, Richmond, VA 23298, USA; dempseys@vcu.edu (S.K.D.); zpdaneva@vcu.edu (Z.D.); ningjun.li@vcuhealth.org (N.L.); pin-lan.li@vcuhealth.org (P.-L.L.); 2 Translational Genomics Lab, Department of Biosciences, COMSATS University, Islamabad 45550, Pakistan; malihazam@gmail.com

**Keywords:** nitric oxide, vasodilation, hypertension, kidney disease, noradrenaline, angiotensin II

## Abstract

The gasotransmitters are a family of gaseous signaling molecules which are produced endogenously and act at specific receptors to play imperative roles in physiologic and pathophysiologic processes. As a well-known gasotransmitter along with hydrogen sulfide and carbon monoxide, nitric oxide (NO) has earned repute as a potent vasodilator also known as endothelium-derived vasorelaxant factor (EDRF). NO has been studied in greater detail, from its synthesis and mechanism of action to its physiologic, pathologic, and pharmacologic roles in different disease states. Different animal models have been applied to investigate the beneficial effects of NO as an antihypertensive, renoprotective, and antihypertrophic agent. NO and its interaction with different systems like the renin–angiotensin system, sympathetic nervous system, and other gaseous transmitters like hydrogen sulfide are also well studied. However, links that appear to exist between the endocannabinoid (EC) and NO systems remain to be fully explored. Experimental approaches using modulators of its synthesis including substrate, donors, and inhibitors of the synthesis of NO will be useful for establishing the relationship between the NO and EC systems in the cardiovascular and renal systems. Being a potent vasodilator, NO may be unique among therapeutic options for management of hypertension and resulting renal disease and left ventricular hypertrophy. Inclusion of NO modulators in clinical practice may be useful not only as curatives for particular diseases but also for arresting disease prognoses through its interactions with other systems.

## 1. Nitric Oxide Background and Production

Nitric oxide is produced from l-arginine and oxygen in a reaction catalyzed by the enzyme nitric oxide synthase (NOS). There are three isoforms of NOS, endothelial (eNOS), neuronal (nNOS), and an inducible NOS isoform (iNOS) [1]. Both eNOS and nNOS are reported to be present in left ventricular myocytes, while nNOS is the myocardial constitutive isoform responsible for the NO-mediated myocardial inotropy and relaxation [2]. Literature-reported differences between eNOS and nNOS [3,4,5] in the subcellular localization (sarcoplasmic reticulum vs. caveolar membrane), mode of activation (via phosphorylation and Ca^2+^ for eNOS and Ca^2+^ for nNOS), and rate of NO production (in nmol of NO min^−1^: 16 for eNOS vs. 96 for nNOS) between eNOS and nNOS may account for their diverse effects on the myocardium. NO exerts its main physiological and pharmacological effect, smooth muscle relaxation, by activating the NO/cGMP pathway. Inhibition of NOS has been shown to result in reduced blood flow in humans [6,7,8] and increased blood pressure and vasoconstriction in animals [9]. This indicates that resistance vessels are under the tonic influence of the vasodilator effect of NO. Nitric oxide is usually measured as the sum total of nitrate (NO_3_^−^) and nitrite (NO_2_) content in the plasma [10,11]. The mechanism of NO production depends on the action of eNOS on l-arginine in endothelial cells.

**Figure 1:**
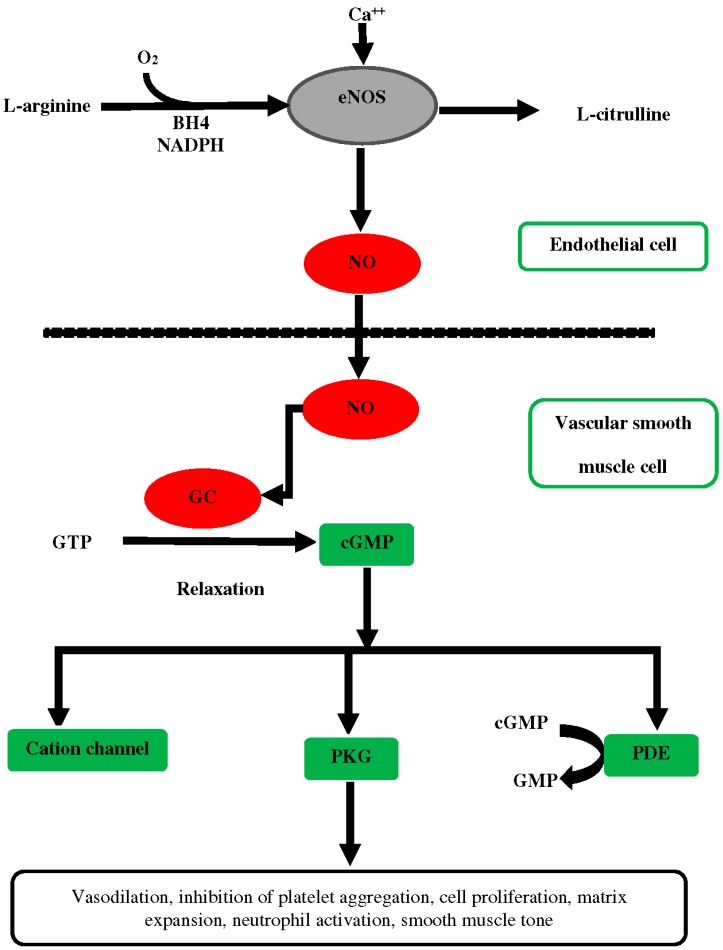
Synthesis of nitric oxide from precursor l-arginine in endothelial cells and mechanism of vasodilation in vascular smooth muscle.

This enzymatic conversion of arginine to NO by eNOS requires oxygen and the reduced cofactors, tetrahydrobiopterin (BH4) and nicotinamide adenine dinucleotide phosphate (NADPH) [12]. Increased intracellular calcium in response to vasodilator agonists such as acetylcholine and bradykinin leads to activation of eNOS and increased production of NO in endothelial cells. NO diffuses into vascular smooth muscle cells where it activates soluble guanylyl cyclase (sGC), in turn forming cyclic guanosine monophosphate (cGMP) to elicit vasorelaxation, as shown in Figure 1. This NO/sGC/cGMP pathway has shown a spatial distribution in the kidney, and its importance in this organ is supported by many studies [10,13]. The level of cGMP is determined by the balance between the synthesizing enzyme soluble guanylyl cyclase (sGC) and catabolizing enzyme (PDE), which metabolizes cGMP to its biologically inactive metabolite, 5′-GMP, as shown in Figure 1. In most cells, the rate of synthesis of cGMP is 10-fold lower than its rate of hydrolysis by phosphodiestrase (PDE) [14]. Therefore, inhibition of PDE is considered a therapeutic target in many pathological conditions such as erectile dysfunction [15], in which an elevated levels of NO is desired. Another interaction of cGMP is with protein kinase G (PKG), which is activated upon _C_GMP stimulation to phosphorylate different target proteins upon cGMP stimulation. These proteins are involved in vasodilation, neutrophil activation, smooth muscle tone modulator, and matrix expansion [13] as shown in Figure 1.

It is also clear that sGC enzyme represents an attractive target for therapeutic intervention in renal diseases featuring NO insufficiency. Any agent capable of activating sGC in a selective manner may have therapeutic potential for such diseases. Among known sGC activators, YC-1 (3-(5′-hydroxymethyl-2′-furyl)-1-benzylindazole) was identified as a moderate NO-independent stimulator of sGC activity by a factor of up to 10-fold [16] that works synergistically with NO via PDE inhibition [17].

In 1994, Stasch and colleagues screened nearly 20,000 compounds and discovered the 5-substituted-2-furaldehyde-hydrazone, a subclass of high-affinity analogues of YC-1. These derivatives are considerably more potent in sGC stimulation and do not appear to inhibit PDE activity, thus offering significantly greater efficacy and selectivity [18]. The heme-dependent sGC stimulators, exemplified by Bay 41-2272 and Bay 41-8543, activate purified enzymes in a fashion synergistic with NO and require the presence of reduced prosthetic heme. Bay 41-2272 (5-cyclopropyl-2-[1-(2-fluorobenzyl)-1*H*-pyrazolo (3, 4-b) pyridin-3-yl]-pyrimidin-4-ylamine) stimulated sGC up to a 25-fold in an NO-independent and heme-dependent manner by interacting with cysteines 238 or 243 of the α1 subunit [18]. In 2002, using a Chinese hamster ovary cell line, BAY W 1449 was identified after screening more than 900,000 compounds [19]. The first NO and heme-independent sGC activator, Bay 58-2667, was selected from about 800 BAY W 1449 analogues, indicating a novel mechanism of enzyme activation [19].

These sGC activators have some advantages over NO precursors, like l-arginine, in clinical practice: (1) they do not lead to the endogenous formation of harmful products (l-arginine: peroxynitrite involving inducible NOS and NO donor: superanion); (2) they show no tolerance like NO donors; (3) they increase sGC activity at low NO concentrations and thus would be very effective with insufficient NO production [13]; and (4) they amplify the cGMP signal exactly at that subcellular location where it is naturally generated and the further cGMP effector pathways are already lined up, thus avoiding the potentially deleterious actions of the effector pathway of NO and the side effects of nitration and nitrosation of the NO donor. S-nitrosation/denitrosation of NO produced by exogenous precursors [20,21] is the major issue in clinical practice. Cardiovascular diseases like hypertension, atherosclerosis, cardiomyopathy, thrombosis, and metabolic diseases have oxidative stress in their pathogenesis. RSNO as a new therapeutic class aimed at improving vascular dysfunctions and overcoming many drawbacks associated with actual NO donors. This is demonstrated by (i) the actual intense investigation on (S-nitrosthiol) RSNO interaction with proteins leading to S-nitrosation, a translational pathway of main importance in cell signaling leading to physiological action such as vasorelaxation and (ii) by many clinical trials based on the administration of S-nitrosoglutathione (GSNO), a physiological RSNO [20].

## 2. Role of Nitric Oxide in Hypertension

A large body of evidence supports that the NO system plays a critical role in blood pressure regulation. The data obtained in the cardiovascular system are consistent, and the literature has described in detail the role of l-arginine (NO donor) in hypertension [22,23]. Reduced concentrations of NO in plasma have been observed in patients with essential hypertension [24], and endothelium dependent vasodilation is impaired in patients with essential hypertension [24,25]. NO bioavailability can be improved by both nonpharmacological and pharmacological approaches. Physical exercise is a nonpharmacological approach to enhance NO bioavailability and improve endothelial function in hypertensive patients [26]. Restoration of eNOS function through augmentation of its substrate, l-arginine, or cofactors for its synthesis has been demonstrated to have beneficial effects. For example, chronic oral supplementation of tetrahydrobiopterin prevented the blood pressure increase in rats with 5/6 nephrectomy [27] and arterial stiffness in fructose-fed insulin-resistant rats [28]. The critical role of eNOS and nitric oxide in the prevention of hypertension is supported by the finding that mice lacking a functional eNOS gene develop hypertension [29]. NG-monomethyl-l-arginine (l-NMMA) has been frequently employed to block the NO production pathway to study the importance of this mechanism in hypertension. Intra-arterial infusion of this inhibitor in untreated hypertensive patients resulted in an abnormal basal nitric oxide-induced vasodilation in the forearm arteriolar bed [30]. Intravenous injection of a low dose of l-NMMA in Sprague-Dawley rats affected the renal excretions of sodium and water without altering blood pressure, but at a high dose it induced hypertension [31].

Supplementation with l-arginine has been a common approach for testing the effect of NO enhancement on hypertension. By providing a source of nitrogen for synthesis of NO by NOS, l-arginine is thought to lower pressure by enhancing vasodilation of resistance vessels indirectly by augmenting the production of NO from NOS. This vasodilator action of NO (endothelium-derived relaxing factor; EDRF) depends on activation of sGC which converts guanosine triphosphate to its product, cyclic 3′, 5′-guanosine monophosphate (cGMP). It has been reported that the impaired nitric oxide production pathway may lead to the onset of essential hypertension [32]. l-arginine and l-citrulline, which is converted to l-arginine, increased the production of NO and prevented the development of salt-sensitive hypertension in Dahl/Rapp rats [33]. In patients with hypertension, the oral administration of l-arginine was an effective therapeutic option [34]. In some studies, vasorelaxation in response to nitroglycerin was also blunted [35], indicating associated changes in responsiveness to NO either supplied exogenously or produced endogenously. Despite several lines of evidence, demonstrating antihypertensive actions of l-arginine, there is a paucity of evidence regarding the role of l-arginine in hypertension. It has been reported that newly diagnosed, mild to moderate hypertensive patients given l-arginine (2 g three times per day) had reduced blood pressure and improved vascular function after one week of treatment [36]. Similarly in patients with mild hypertension, the infusion of l-arginine (500 mg/kg for 30 min) lowered the mean blood pressure by 8% and reduced the renovascular resistance [37]. l-arginine reduced serum endothelin-1 and angiotensin II levels [38] which may be contributory factors in the lowering of blood pressure. Overall, these data support the idea that the endothelium plays an important role in the modulation of blood pressure and that NO is a key component responsible for this modulation and is generally known as EDRF. It may be possible to pharmacologically modulate the endogenous production of NO by exogenous agents to reduce blood pressure.

The upregulation of the NO/sGC/cGMP pathway in arterial hypertension has been identified as a promising therapeutic goal for lowering blood pressure and reducing associated complications related to heart and kidney function based on experimental models of hypertension and NOS-inhibition without causing tolerance [18,19,39].

## 3. Role of Nitric Oxide in Left Ventricular Hypertrophy (LVH)

Although nitric oxide has been a gasotransmitter of great interest for hypertension, investigations of its role in the pathophysiology or treatment of left ventricular hypertrophy (LVH) have lagged. However, findings resulting from aggressive research in hypertension may be relevant for the treatment of LVH. LVH is one of the major consequences or comorbidities of hypertension [40,41,42]. Some evidence supports an antihypertrophic role for nitric oxide in LVH as a result of essential hypertension [43]. In rats with hypertension induced by the NO synthase inhibitor, N^G^-nitro-l-arginine methyl ester (l-NAME), l-arginine treatment reversed several markers associated with LVH [44]. Several investigations have studied LVH in combination with essential hypertension [45,46,47,48], but the results regarding the regression of LVH were not encouraging. However, in spontaneously hypertensive rats (SHR), a 12-week treatment with l-arginine ameliorated LVH without affecting arterial blood pressure [46]. In contrast, another study showed that treatment of salt-sensitive Dahl-Rapp hypertensive rats with l-arginine reduced blood pressure to almost normotensive levels [33]. Few studies, however, have accounted for the fact that attenuation of LVH may be time-dependent as there was no regression of LVH either in SHR or in stroke-prone SHR by chronic administration of l-arginine for 6 weeks and one month, respectively [47,48]. 

l-arginine has been investigated extensively in animal models and humans for its effects on hemodynamic and metabolic changes, and other cardiovascular complications of hypertension [44,49,50,51]. In the cited study showing reversal of LVH in rats with l-NAME-induced hypertension and LVH, it was noted that the effects were dependent on blood pressure reduction but independent of restoration of NOS activity [44]. A large amount of evidence has shown that NO has antihypertrophic effects in the heart of SHR after chronic treatment with l-arginine [46], but these effects were independent of blood pressure-lowering. A similar response was observed in eNOS-overexpressing mice, which exhibited an attenuated cardiac hypertrophic response to chronically administered isoproterenol [52]. These studies confirmed NO as a negative modulator of heart hypertrophy in different models.

As l-arginine may enhance the production of NO by either eNOS or nNOS [1], it cannot be assumed that eNOS is the mediator of the beneficial effects of l-arginine in LVH. It is documented that mice with deficiency of either nNOS and eNOS developed spontaneous cardiac hypertrophy, and that animals with absence of both nNOS and eNOS developed more severe hypertrophy [53]. 

Currently, few studies have investigated the role of NO in dedicated models of LVH, although previously the role of the NO donor, l-arginine in the hypertensive model of LVH has been investigated [54,55]. There are contradictory findings in the LVH in SHR data where chronic treatment with l-arginine attenuated the LVH in SHR independent of blood pressure [46], but in salt-sensitive SHR, chronic treatment with l-arginine decreased the blood pressure to a normotensive level [33]. In studies using SHR and stroke-prone SHR [48], no LVH attenuation was observed after chronic administration of l-arginine intraperitoneally for 6 weeks or in drinking water for one month. While enhancement of the eNOS/NO pathway in myocardium by l-arginine treatment undoubtedly contributed to the mechanism of amelioration of LVH in Wistar-Kyoto rats [56], its enhancement at other sites is most likely beneficial. Notable in this regard is the kidney, where l-arginine treatment also improved renal cortical blood flow and sensitized α_1_ adrenergic receptors [56]. It may be speculated that upregulation of eNOS/NO/cGMP was important to maintain heart architecture and physiology which will not only control the mortality but also prognosis of the disease.

In summary, NO is a well-known and established antihypertrophic agent which arrests the progression of LVH by lowering blood pressure and interacting with other systems like renin angiotensin aldosterone system (RAAS) and sympathetic nervous system (SNS), which will be addressed further below in this review.

### Alteration of Expression of Endothelial Nitric Oxide Synthase (eNOS) in LVH

Endothelial nitric oxide synthase (eNOS) is considered the most physiologically relevant NOS isoenzyme in the heart and is constitutively expressed in both the endocardium and in the cardiomyocytes of the myocardium [1,57]. Significant work has been done to investigate the role of eNOS in the pathophysiology underlying LVH using different model systems. In mice with LVH induced by transverse aortic constriction (TAC), eNOS deficiency resulted in enhanced LVH and fibrosis compared to wild type (WT) mice [58]. Early observations showed cardiomyocyte-restricted restoration of eNOS activity blunted LVH and dysfunction induced by transverse aortic constriction (TAC) mice [59]. Another study demonstrated that the presence of NOS3 (eNOS) limited LV dysfunction and remodeling in a murine model of MI by an afterload-independent mechanism [60].

The beneficial role of eNOS expression in cardiomyocytes remains to be a topic of debate. It was observed that sustained pressure overload induced by constriction of the thoracic or abdominal aorta resulted in greater LV dysfunction in eNOS^−/−^ mice than in wild type mice [61,62]. In contrast to these findings, it was reported that eNOS deficiency preserved the morphology and function of LV after the transverse aortic constriction (TAC) procedure as compared to wild type mice [63], whereas other studies have reported that eNOS of bone marrow plays a beneficial role in amelioration of cardiac hypertrophy [58].

While the above highlighted literature clearly supports roles for NO in LVH and cardiac remodeling, after more than 25 years of research on NO, the story for NO and its role in cardiac pathophysiology seems to be multifolded. Whether this relates to the complexity of the NO system in the heart or the presence of multiple NOS isoforms in multiple cell types remains to be determined. Further research is clearly required to unfold the dual-faced nature of eNOS in cardiac hypertrophy and dysfunction.

## 4. Roles of Nitric Oxide in the Kidney

Nitric oxide, being a vasodilator, is considered to play a significant role in the homeostatic regulation of renal hemodynamics in both normotensive and hypertensive states [31]. NO mainly produces vasodilation by the eNOS/NO/cGMP pathway. In glomeruli, eNOS is located in endothelial cells, whereas nNOS is found in macula densa. NO deficiency is considered critical for the production of matrix and its accumulation in the kidney [64,65] and documented in various experimental renal disease model such as diabetic and hypertensive nephropathy, obstructive nephropathy, glomerulosclerosis, and renal diseases due to cyclosporine A and radiocontrast [66,67,68,69,70]. It is clear from this literature that NO deficiency and downregulation of the NO/sGC/cGMP pathway are commonly observed in renal diseases. In addition, a deficiency of renal NO production has been proposed as a main mechanism of development of systemic hypertension, which may be attributed to attenuation of its beneficial effects on renal sodium and fluid excretion and resistance of the renal vasculature [31]. Existing evidence that deficiency in NO interferes with the reabsorptive and excretory functions of the kidney opens windows for investigations of the underlying mechanisms for these processes. It is reported that in chronic renal failure, there is an accumulation of N^G^,N^G^-dimethylarginine (asymmetric dimethylarginine, ADMA) which leads to impaired NO production, hypertension, and immune system dysfunction [71]. Existing data suggest that NO regulates many aspects of normal renal homeostasis, and any abnormality in endogenous NO production may lead to nephropathy. Exogenous supplementation of l-arginine ameliorated the progression of renal disease in rats with subtotal nephrectomy [72]. An inhibitor of NO, *N*-methyl-l-arginine, increased the renal sympathetic nerve activity in rats [73]. It supports the contention that augmentation of the endogenous NO supply dampens renal sympathetic nerve activity, leading to increased blood flow to the kidney. In chronic renal failure, the NO concentration in plasma was reduced and exogenously supplied l-arginine reversed the conditions. It is reported that captopril administered in combination with l-arginine prevented chronic renal failure by an l-arginine-NO dependent pathway [74]. These evidences collectively strengthen the hypothesis that NO is protective against different injuries to the kidney. In ischemia, kidney function was sustained by EDRF/NO and inhibition of NO synthase increased sensitivity to damage [75]. It has been reported that greater vulnerability to ischemia of diabetic kidney is associated with decreased production of and response to NO [76]. The production of NO in the kidney appears to be important for regulation and protection of many kidney functions. For example, NO is reported to control the functions of the proximal convoluted tubule, an important segment for reabsorbing 65–70% of Na^+^ and water reabsorption and play a fundamental role in their physiology and pathology [77].

## 5. Interaction with Alpha Adrenergic Receptors of the Kidney

### Renal α_1_-Adrenergic Receptors Subtypes

Both the α_1_-adrenoceptors and NO are operated by G-proteins, and it can be assumed that modulation of one can translate similar changes to the other. There is extensive literature regarding the roles of NO in the heart and kidneys and in hypertension, but few studies conducted so far have explored the role of NO on α_1_-adrenoceptors. In many diseases like renal failure [78], in fructose-fed animals [79] and diabetic hypertensive rats [80], α_1_-adrenoceptors were reported to show reduced responsiveness to an adrenergic agonist, but none of the studies attempted to modulate this responsiveness or resensitize α_1_-adrenoceptors using NO. The regional distribution of α_1_-adrenoceptors in the rat kidney has been reported [81], with the highest density in the cortex, decreasing towards the papilla. Furthermore, α_1A_ and α_1B_ are present in equal density in the cortex and medulla, whereas in proximal convoluted tubules, three subtypes (α_1A_, α_1B_, and α_1D_) appear equally distributed. Studies support that α_1A_ adrenoreceptors are functionally dominant in adrenergic-mediated vasoconstriction in undiseased conditions [82,83,84] as well as in pathologic states such as two-kidney, one-clip Goldblatt hypertension, deoxycorticosterone acetate (DOCA)-salt hypertensive rats [85], diabetic one-clip Gold blatt hypertensive rats [86], and fructose-fed Sprague-Dawley rats [87]. Whether shifts in the functional behavior or composition of α_1_-adrenoreceptor subtypes occur in the kidney vasculature under pathological conditions has been of interest. One study found that the adrenoreceptor subtype composition in the renal vasculature of spontaneously hypertensive rats was unaffected by diabetic nephropathy in the presence of hypertension [80]. Another study, however, found that induction of renal failure in hypertensive rats led to a greater functional role of α_1B_ adrenoreceptors [78]. Vasoconstriction induced in the failed kidney was mediated by α_1B_-adrenoreceptor and appeared independent of the hypertension. Similarly, the long term consumption of a high salt diet in Wistar-Kyoto rats also shifted the vasoconstriction responses toward α_1B_ adrenoreceptor mediation [88]. Collectively, it can be speculated that α_1B_ is functionally dominant in maintaining the renal vasoconstriction in response to adrenergic agonist as compared to α_1A_ adrenoreceptor. In vivo and in vitro studies have reported that α_1D_ adrenoreceptors are involved in mediating renal vasoconstriction in resistance vessels along with α_1A_ adrenoreceptor in normal states [83,84,88], essential hypertension along with cardiac failure [89], essential hypertension with diabetic nephropathy [80], essential hypertension with renal failure [78], one clipped Gold blatt hypertension [86], fructose fed rats [79], and in LVH induced by isoprenaline and caffeine [90].

The first piece of evidence for interaction of α_1_ adrenoreceptors and NO precursor was reported only recently [10]. The findings suggested that exogenous NO precursor upregulated the renal eNOS/NO/cGMP pathway in LVH rats and resulted in augmented responsiveness of α_1A_, α_1B_, and α_1D_ adrenoreceptors to adrenergic agonists. Previous reviews have described the importance of this pathway in the kidney [10,13]. This NO/sGC/cGMP pathway has been found to exhibit a specific spatial distribution. In glomeruli, eNOS is located in endothelial cells, whereas nNOS is found in macula densa. NO diffuses to sGC in glomerular arterial walls and mesangial cells [91]. The activated sGC increases the levels of cGMP which increases its downstream effects via PKG. In glomeruli, protein kinase G (PKG) is present in smooth muscle and mesangial cells [92]. Heterogeneous distribution of eNOS and sGC and uniform distribution of sGC and PKG suggests that NO acts through elevation of cGMP, a second messenger of the G-protein pathway. It is reported that hyperactivation of sympathetic nervous system in LVH [93] down regulated α_1_-adrenoreceptors [91]. The α_1_-adrenoreceptors and NO both are operated by G-proteins makes it fair to speculate that NO precursor upregulated eNOS/NO/cGMP pathway and increased the responsiveness of α_1_-adrenoreceptors by modulation of G-protein.

Another role played by NO in modulation of α_1_-adrenoreceptor sensitization to adrenergic agonists is by S-nitrosylation of β-arrestin [94] which is responsible for signaling from G-protein coupled receptors and is involved in receptor desensitization and downregulation via internalization [95]. Beta-arrestin binds to activated G protein-coupled receptor kinase-phosphorylated receptors, which leads to their desensitization with respect to G proteins [95,96]. S-nitrosylation of β-arrestin is also considered to control the β adrenergic signal trafficking [97]. The literature supports a strong connection between β-arrestin and α_1_-adrenoreceptors [94,96], while at the same time literature showed connection between NO, β-arrestin, and α_1_-adrenoreceptors [94,97].

Further studies are required to understand the action of NO precursor on the sensitization of α_1_-adrenoreceptors in different pathological conditions dependent on either G-protein coupled receptors or β-arrestin. Whether upregulation of the renal eNOS/NO/cGMP pathway in LVH rats results in augmented α_1A_, α_1B_, and α_1D_ adrenoreceptors responsiveness is another area of interest that needs to be addressed.

## 6. Interaction of Nitric Oxide with Other Systems 

### 6.1. Nitric Oxide with Angiotensin II

Angiotensin (Ang) II is a potent vasoconstrictor [98] that plays a significant role in the pathogenesis of cardiovascular diseases including hypertension [99], cardiac hypertrophy [100], and acute kidney injury [101]. Depending upon the spectrum of Ang II-induced pathologies, it became an obvious therapeutic goal either to reduce the elevated levels of Ang II or counteract the effects produced by Ang II to treat diseases of cardiovascular and renovascular system. The counteracting effects of endogenous NO on Ang II in the cardiovascular system have been important points of interest for researchers. The balance between endogenous levels of NO and Ang II appears to be a key factor in the various pathologies of the cardiovascular system. It has been observed in vivo that intravenous administration of subpressor doses of Ang II in anesthetized rats increased the tissue concentration of NO in the renal medulla as compared to renal cortex, while l-NAME infusion in the medulla blocked Ang II-induced NO in the medulla but not cortex [102]. It appears from published data that pressor doses of Ang II cause vasoconstriction in the kidney, and that a compensatory mechanism releases NO to buffer the action produced by Ang II. Similarly, NO has been reported to play a renoprotective role in the chronic angiotensin-induced model of hypertension [103]. Ang II levels were found to be elevated in LVH [104], while administration of exogenous l-arginine inhibited the progression of LVH by increasing plasma and myocardium levels of NO [56]. It is known that Ang II levels are increased while NO levels are reduced in LVH, and LVH resulted in decreased responsiveness of α-adrenergic receptors to adrenergic agonists [90,105]. Administration of the exogenous NO precursor upregulated the renal eNOS/NO/cGMP pathway in LVH rats and reduces the levels of Ang II which resulted in augmented responsiveness of the α_1A_, α_1B_, and α_1D_ adrenoreceptors to the adrenergic agonists [10]. This augmented responsiveness of the α_1A_, α_1B_, and α_1D_ adrenoreceptors to the adrenergic agonists may be multifaceted but one of the factors is counteraction of NO to the effects produced by Ang II in LVH on adrenoreceptors. The equation of NO and Ang II is well balanced, and inhibiting or suppressing one heightens the activity of the other. In the renovascular system, it has been reported that renal vasodilator response to l-arginine was abolished in untreated essential hypertension, while the same responses were restored when the hypertension was treated with angiotensin converting enzyme (ACE) inhibitors [106].

**Figure 2:**
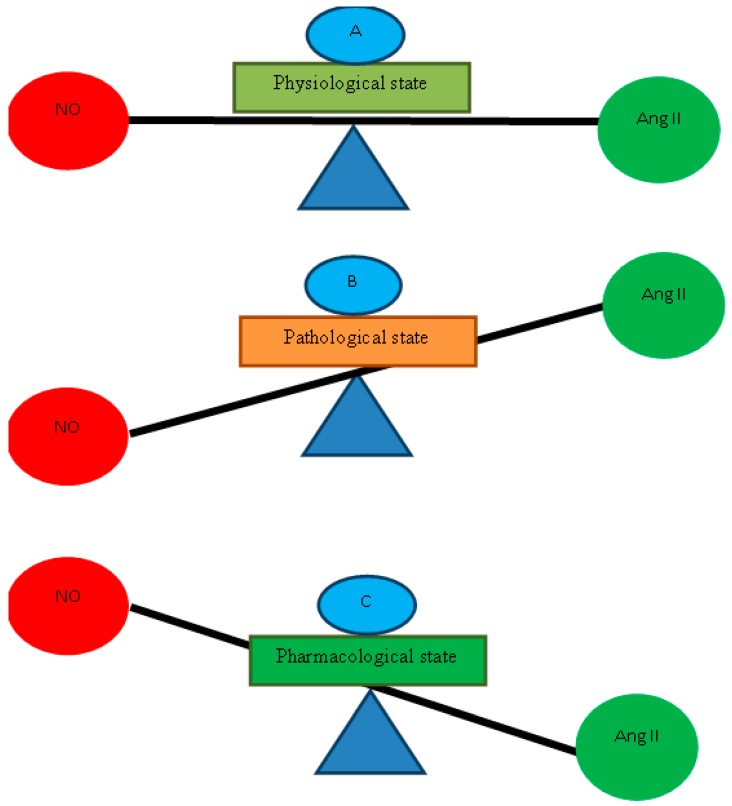
Different levels of NO and Ang II depicting different states of the body. (**A**) shows normal levels of NO and Ang II in normal physiological conditions. (**B**) represents that levels of Ang II increased while that of NO is reduced in plasma and tissue in pathological states like hypertension and LVH, while (**C**) indicates that pharmacological treatment with NO donors or Ang II inhibitors increases the NO and reduced the levels of Ang II to offset the pathological state.

Both NO and Ang II play a crucial and surrogate role in the pathogenesis of hypertension. It is reported that chronic inhibition of NO synthesis resulted in a severe form of hypertension which may lead to the activation of renin–angiotensin vasoconstrictor system [107]. In conscious rats, chronic inhibition of NO by l-NAME resulted in sustained hypertension while blockade of AT1 receptor produced little effect on blood pressure (BP), blockade of α adrenergic receptors resulted in moderate fall in BP while combined blockade of AT1, and α adrenergic receptors produced profound drop in pressure indicate that chronic inhibition of NO, hypertension is due to combined stimulation of AT1 and α adrenergic receptors [108]. Literature provides evidence that NO is not only involved in modulation of Ang II activation, but there is supporting evidence that NO counterbalances the vasoconstriction produced by Ang II by increasing vasodilator tone [109]. The absence of vasodilator tone may be a reason for Ang II induced hypertension as NO has been reported to oppose the Ang II induced arterial pressure in treadmill running among normal rats and rats with heart failure [110]. Elevated levels of Ang II in plasma and tissue are not considered as beneficial for human physiology. Increased Ang II indicates an underlying cause which needs to be diagnosed. This condition could be alleviated by using NO donors which can offset the effects produced by Ang II. This scenario is explained in Figure 2. Several transmembrane-spanning receptors and their downstream signaling molecules could be modified by NO, including β arrestin [97] and Ang II receptors (AT_1_R), regulate β-arrestin bindings to β adrenergic receptors by heterodimerization [111] and calcium channels activation [112]. It could be speculated that exogenous administration of a NO donor modulates β-arrestin which in turn downregulates AT_1_R and antagonizes Ang II effects. AT_1_R regulates desensitization of β-adrenergic receptors by facilitating binding of β-arrestin to β-adrenergic receptors [111]. Another direct pathway for antagonizing the Ang II action by NO is direct effects on AT_1_R. It is reported that excessive exposure of NO downregulates Ang II receptors [113]. The above-mentioned literature emphasizes the pharmacological and physiological interaction of NO with Ang II not only in the activation of Ang II, but also in counterbalancing the effects produced by Ang II in cardiovascular and renovascular system either blocking β-arrestin or direct action on AT_1_R. 

### 6.2. Nitric Oxide with Gaseous Transmitters Like Hydrogen Sulfide

**Figure 3:**
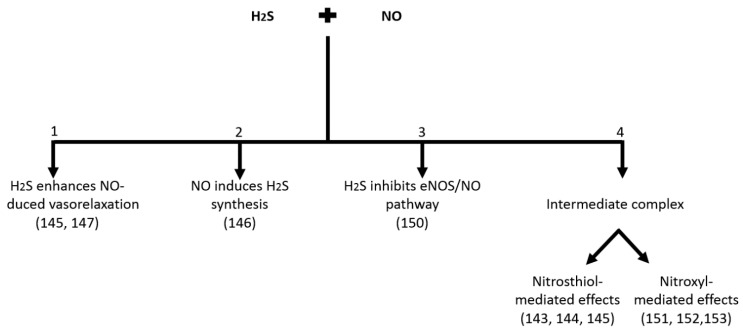
Overall summary of interactions described between H_2_S and NO in living system.

The family of endogenous gaseous mediators is comprised of nitric oxide (NO), hydrogen sulfide (H_2_S), and carbon monoxide (CO) [114]. H_2_S [115] has attracted great interest, especially with regard to its interplay with NO [116,117,118,119] (Figure 3). These two mediators produce vasodilation by a mechanism resulting in increased cyclic guanosine monophosphate (cGMP). The dependence of the effects of H_2_S on _C_GMP has been a matter of great debate and several studies have conflicting results. Early studies using functional approaches to test the contribution of cGMP to the relaxing effects of H_2_S which produced mixed results. It was reported that relaxation of the aorta by H_2_S was independent of cGMP [120]. Relaxation to H_2_S in the mouse gastric fundus and inhibition of platelet aggregation were reported to occur independently of cGMP [121]. In contrast to this finding, effects of sodium hydrogen sulfide on Mercenaria gills brachial muscles were reduced by sGC/PKG inhibition [122], which suggest the relaxation of H_2_S is dependent on the sGC/PKG pathway. These mixed findings concerning hydrogen sulfide′s dependence on _C_GMP are explained in a review article [123].

In this section, this review article focuses on the interaction of H_2_S and NO. Each has been observed to stimulate the synthesis of the other and proposed as a surrogate marker for production of the other. Discrepancies in some interaction studies suggest the formation of an intermediate molecule, although this has remained a contentious issue [118,124,125]. There are two different schools of thought regarding the interchangeable production of H_2_S and NO. In 1997, it was shown that low concentrations of H_2_S (<30 μM) greatly enhanced the NO-induced relaxation of smooth muscle by 13-fold [126]. This finding was significant because a synergistic response between H_2_S and NO may be a desirable and beneficial therapeutic outcome for treatment of hypertension. This group further elaborated that the enhancement of NO-induced vasorelaxation was specific for H_2_S, but that NO could not potentiate the vasorelaxant effect of H_2_S. In contrast, another study [127] suggested that NO dose-dependently (1–100 μM) upregulated the production of H_2_S in rats. Two potential mechanisms of upregulated production of H_2_S by NO were suggested by the authors: (i) NO increases cystathione γ lyase (CSE) activity which ultimately leads to the production of H_2_S, and (ii) NO increases the activity of a protein kinase that is dependent on cGMP and leads to increased CSE protein.

Another growing body of evidence supports a role of H_2_S as a cofactor required for the generation of NO from nitrite [128]. Grossi et al. [129] documented that interaction between hydrogen sulfide and nitric oxide resulted in the formation of the intermediate compound, S-nitrosothiol, which released NO (via hemolysis) (Figure 3). Later, a mechanism of NO production from sodium nitroprusside (SNP) was proposed [130]. In 2006, Whiteman et al. [125] proved that the interaction between the two gasotransmitters resulted in the formation of nitrosthiol and a small portion of NO. 

During the debate regarding this interchangeable production of H_2_S and NO, another study illustrated that H_2_S was responsible for the direct inhibition of eNOS [131] (Figure 3) and that it was responsible for the production of NO in endothelial cells. In later work, Kubo et al. [131] elaborated that H_2_S was not only responsible for inhibition of eNOS but also iNOS and nNOS.

It was reported that the intermediate complex formed due to interaction between H_2_S and NO does not have a vasorelaxant effect [124]. Another area of interest is the chemical interaction between two gasotransmitters, H_2_S and NO, resulting in formation of the intermediate, S-nitrosothiol [125,126], which is responsible for its vasorelaxant effect on the blood vessels. The presence of this intermediate molecule has not been fully confirmed and considered as possible molecule for said action. It has been previously [124] stated that the intermediate molecule is indeed S-nitrosothiol, but that has no vasorelaxant effect in vivo and in vitro. One school of thought reported that the interaction between NO and H_2_S leads to the formation of the nitroxyl group [132], as shown in Figure 3, and this nitroxyl molecule had positive inotropic and vasodilation activities [133]. These results strengthen the evidence that nitroxyl/NO produce positive inotropic and lusitropic effects (independent of β-adrenergic stimulation) in the failing heart [134].

### 6.3. Nitric Oxide with Noradrenaline

**Figure 4:**
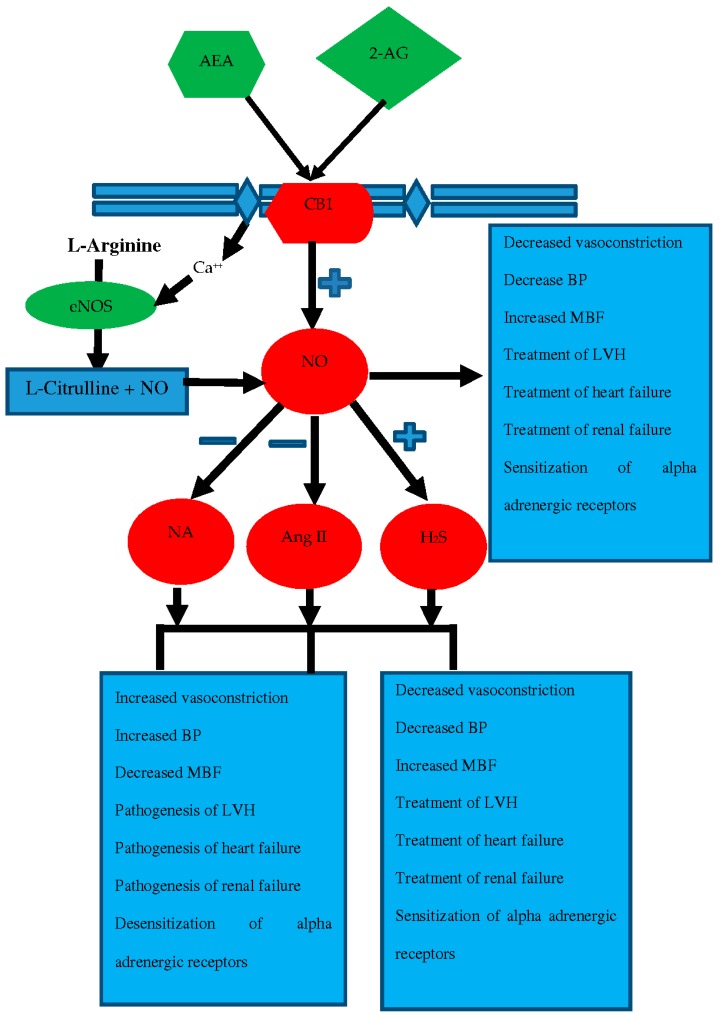
Interactions of NO with different systems and modulation of their functions in cardiovascular and renovascular systems.

Similar to its interaction with angiotensin, nitric oxide may be expected to interact with noradrenalin (NA), another major vasoconstrictor system in the body. The sympathetic nervous system (SNS) modulates the tone of arteries, vascular beds, and adrenergic receptors to control blood pressure. NA levels in the plasma are usually taken as a marker of the SNS activity [90,135]. NA is a sympathomimetic neurotransmitter which effects to increase blood pressure are ascribed to its agonist activity at adrenergic receptor [136]. LVH is associated with reduced levels of NO and elevated levels of NA [56] similar to Ang II. l-arginine, the precursor NOS substrate, reversed endpoints associated with LVH by increasing NO and lowering plasma levels of NA and Ang II [56], indicating the buffering role of NO against NA and Ang II. Although a direct relationship between NA and NO has not been studied, arguments can be made that the absence of a vasodilator like NO either in plasma or tissue allows a counteracting vasoconstrictor substance to dominate and this can be true vice versa. It can be explained that inhibition of NO synthesis by l-NAME has a dual effect on perfusion pressure in that vasodilation (direct effect of NO) is reduced and at the same time vasoconstriction is increased (effect of increased norepinephrine) [137]. Essential hypertension [138] and LVH [139] are each associated with higher sympathetic activity and elevated NA levels and reduced NO levels, while NO donor treatment reversed these changes in hypertension [140] and LVH [56]. In addition to being a direct vasodilator, NO also alters vascular reactivity at sympathetic neuroeffector junctions, such as the rat mesenteric bed, by deactivating the vasoconstrictor, norepinephrine [137]. This association between NO and NA argue that responses produced by NA are antagonized by NO while their effect on each other’s production was still unfolded. Interrelated production can be explained by a study that depicted the mutual modulation between NE and NO during the immune response of scallop hemocytes against lipopolysaccharides (LPS) [141]. In this study, NE was involved in the modulation of NO generation, while in reverse, NO might also play a role in feedback regulation of NE concentration. As mentioned earlier, the absence of NO can heighten the vasoconstriction phenomenon by increasing the levels of vasoconstrictors like NA and Ang II. This point could be explained by a prolonged blockade of nitric oxide synthesis that causes arterial hypertension (associated with an activation of the sympathoadrenal system) [142]. Nitric oxide is not only involved in functional antagonism of NA, but also affects the production of NA. Some studies have reported that NO plays a crucial role in the modulation of catecholamine release at peripheral sympathetic nerve terminals [143,144]. Nitric oxide may also modulate the release of NA at some blood vessels [145] and exert negative control over the evoked NE release from sympathetic nerve fibers innervating the rat heart [146]. Functional antagonism by NO on NA is demonstrated in Figure 4. The eNOS-derived NO may alter the sympathetic nerve release of NE, as research investigating the profound interaction between NO and NE could demonstrate that eNOS-derived NO inhibits the NE release in neural/vascular tissues, thereby effectively diminishing sympathetic tone [146,147], as shown in Figure 4.

It may be concluded from the above literature that the net balance between circulating levels of NO and NA defines the contraction and relaxation of arteries and vessels to maintain normal physiological functioning. The imbalance between NO and NA yields to a pathological state in cardiovascular and renovascular systems which needs further pharmacological intervention.

### 6.4. Nitric Oxide with Endocannabinoids

The EC system consists of the two main endogenous cannabinoid receptor agonists, anandamide (AEA) and 2-arachidonylglycerol (2-AG), their respective hydrolyzing enzymes, fatty acyl amide hydrolase (FAAH) and monoacylglycerol lipase (MAGL), and the cannabinoid receptors, CB1 and CB2. AEA is synthesized mostly by release from *N*-arachidonoyl phosphatidylethanolamine mediated by *N*-arachidonoyl phosphatidylethanolamine-specific phospholipase D, and its agonist effect on CB receptors is controlled by FAAH-mediated metabolism to inactive arachidonic acid and ethanolamine [148]. In contrast, 2-AG is synthesized from membrane phospholipids by phospholipase C beta and diacylglycerol lipase (DAGL), and it undergoes hydrolysis by MAGL to form arachidonic acid and glycerol [149]. Both EC agonists, AEA and 2-AG, have been a subject of great interest due to their capacities to activate cannabinoid receptors [150]. Proposed relationships and interactions of ECs with NO are shown in Figure 4.

ECs and the prototype cannabinoid and well known drug of abuse, tetrahydrocannabinol (THC), share the ability to elicit a drop in blood pressure and heart rate [151]. Interestingly, hypotension induced by AEA was absent in normotensive rats [152,153] but present in spontaneously hypertensive rats [154], suggesting a sympathoinhibitory action of AEA. This effect may be explained by inhibition of peripheral NA release mediated by AEA-induced NO release [147,155], thereby offsetting the pharmacological responses of NA (Figure 4). AEA can exert an autoregulatory function in vascular endothelial cells by stimulating eNOS and subsequent NO generation via the CB1 receptor [147]. The link between AEA and NO formation has been described in other systems [156]. The previously discussed interaction of NO and NA together with evidence that AEA elicits sympatho-inhibition activity by inhibiting NA release [157,158] clearly supports the triangular connection between AEA/NO/NA pathways shown in Figure 4. This linkage whereby ECs offset the responses of NA by releasing NO is supported by studies in rats [147] and anesthetized dogs [159]. CB1 receptors mediate their responses by releasing NO, and it can be speculated that CB1 agonists increase NO activity. Though the EC system has not been studied as extensively in the kidney as compared to the brain, our laboratory showed exogenous intramedullary administration of AEA produced diuretic and natriuretic effects without impacting medullary blood flow or blood pressure [160]. It may be hypothesized that AEA or other EC ligands for the CB1 receptor mediate their actions in kidney via an NO-dependent mechanism. 

In summary, autoregulatory pathways by which ECs modulate blood pressure are present in higher animals including humans, and these pathways involve complex interactions between NA, NO, and other vasoconstrictor and vasodilator systems [161].

## 7. Conclusions

A large body of evidence supports roles of the vasodilator, NO, as an antihypertensive, antihypertrophic, and renoprotective agent. The linkage of a single molecule with such a diverse number of physiological systems in the human body underscores its promising therapeutic potential in cardiovascular and renovascular complications. As a sympatholytic agent, NO not only contributes to the regulation of blood pressure, but also plays a renoprotective role in hypertensive patients by antagonizing the deleterious effects of NA and angiotensin and by mediating the release of another promising gasotransmitter, H_2_S. This review not only highlighted the beneficial roles of NO in the cardiovascular system and kidney, but also emphasized the link between NO and different physiological systems whose effects are either mediated by NO or antagonized by NO.

## Abbreviations

NO	Nitric oxide
NADPH	Nicotinamide adenine dinucleotide phosphate
EDRF	Endothelial derived vasorelaxant factor
ECs	Endocannabinoid system
eNOS	Endothelial nitric oxide synthase
nNOS	Neuronal nitric oxide synthase
iNOS	Inducible nitric oxide synthase
cGMP	Cyclic 3′,5′-guanosine monophosphate
GC	Guanylate cyclase
l-NMMA	NG-monomethyl-l-arginine
l-NAME	N^w^-nitro-l-arginine methyl ester
sGC	Soluble guanylyl cyclase
PDE	phosphodiesterase
SHR	Spontaneously hypertensive rats
LVH	Left ventricular hypertrophy
TAC	Transverse aortic constriction
ADMA	N^G^,N^G^-dimethylarginine
PCT	Proximal convoluted tubules
LPS	Lipopolysaccharide
H_2_S	Hydrogen sulfide
BH4	Tetrahydrobiopterin
NE	Norepinephrine
NA	Noradrenaline
SNP	Sodium nitroprusside
AEA	Arachidonoyl ethanolamine (Anandamide)
2-AG	2-arachidonoylglycerol
FAAH	Fatty acid amide hydrolase
MAGL	Monoacylglycerol lipase
CB1	Cannabinoid receptor type 1
CB2	Cannabinoid receptor type 2
DAG	Diacylglycerol lipase
THC	Tetrahydrocannabinol
